# The Role of Psychology in Media During the COVID-19 Pandemic: A Cross-National Study

**DOI:** 10.5334/pb.1054

**Published:** 2022-04-12

**Authors:** Angélica Caicedo-Moreno, Andrea Correa-Chica, Wilson López-López, Pablo Castro-Abril, Idaly Barreto, Juan Diego Rodriguez-Romero

**Affiliations:** 1 Universidad del País Vasco, Departamento de Psicología Social, ES; 2 Universidad de Santiago de Compostela, Department of Social Psychology, Basic and Methodology, ES; 3 Pontificia Universidad Javeriana, Facultad de Psicología, CO; 4 Universidad Católica de Colombia, Facultad de Psicología, ES

**Keywords:** COVID-19, Psychology, Media, Topic Modeling, Text Analysis

## Abstract

Dealing with COVID-19 and with the preventative measures that have been taken to mitigate the transmission of the virus causing the pandemic has posed a great challenge to the population. While psychologists have expertise with regard to preventive behavior change and to dealing with the mental health impact of measures, their expertise needs to be effectively communicated to the public. Mass media play a critical role in times of crisis, in many cases being the only source of information. While most research focuses on the importance of information content as a factor affecting psychological responses to a collective traumatic event, the way information is framed in the media is likely to influence the way health professionals are perceived as trustworthy. This study aimed to analyze the media framing of information from psychology during the COVID-19 pandemic in six countries from America and Europe, identifying the most recurrent topics in the news (n news items = 541) related to psychology and mental health. In all six countries the media address the psychological needs of the population, which vary depending on the imposed restrictions. The news content is influenced by the scientific sources used by the media. While the most prevalent topics focus on psychological risk and the need to seek mental health care, the least prevalent topics relate to counseling and behavioral guidelines for managing the psychological consequences of the pandemic. The study findings provide insight into how psychological knowledge contributes to the understanding and mitigation of COVID-19 consequences in different countries and identified fields where psychologists were consulted to respond to a health emergency. They also show a preference to consult other experts when searching for contextual or more macro-social explanations of critical situation.

## Introduction

The emergence of the SARS-CoV-2 coronavirus, and of the COVID-19 disease it triggers poses a profound threat to physical and mental health, as well as to social, political, and economic stability worldwide. Very few events in recent years can compare with the impact of the global health crisis caused by COVID-19, of which the social, economic, health, and life consequences have been dramatic (Moya et al., 2020). The events related to the COVID-19 crisis have also received extraordinary media coverage. In January 2020 alone, there were 23 times more news articles regarding COVID-19 when compared to similar news articles about Ebola in its initial phase during 2018 (Masip et al., 2020). Mass media have become the primary source of information for a significant segment of the population to remain updated, make decisions, and modify behaviors to protect their health and safety (Gui, 2021; Krawczyk et al., 2021; Zheng et al., 2020).

Media coverage of the COVID-19 crisis has focused primarily on reporting on virus spread statistics, difficulties in containing contagion, economic impacts, prevention and protection strategies, and impacts on the psychological and social well-being of the measures implemented (Ng et al., 2021). In doing so, media draw heavily on insights from science. Reliance on science has been considered as crucial for compliance with health measures such as social distancing or vaccination (Bicchieri et al., 2021). Since views from scientists can serve as cognitive shortcuts for decision-making on actions that would be costly when made individually (Sturgis et al., 2021), scientific experts in areas such as medicine, virology, epidemiology, or psychology may give information based on their areas of expertise, providing concrete and practical recommendations through the media (Shullman & Evans, 2020, López-López, 2020).

Regarding psychology, international organizations such as the International Union of Psychological Science [IUPsyS], the International Association of Applied Psychology [IAAP], the Interamerican Society of Psychology [SIP], the American Psychological Association [APA], the European Community Psychology Association [ECCP], or national organizations provided information to help the population manage the COVID-19 crisis from the outset. These contributions are of vital relevance in the public health crisis caused by COVID-19. Psychology can contribute with evidence-based knowledge to promote preventive behaviors (e.g., use of masks), coping behaviors (e.g., dealing with confinement), and social support behaviors (e.g., prosocial actions). As such, psychology can provide tools to help mitigate the impacts of the COVID-19 crisis at the individual, interpersonal and collective levels.

The adverse mental health consequences of COVID-19 were evident from the onset of the crisis (Bueno-Notivol et al., 2021; Taquet et al., 2021). Fear of contagion, transformations in individual and social dynamics due to the measures containing the spread of the virus, information saturation, and the economic repercussions associated with the COVID-19 crisis have posed a risk to mental health globally (Garfin et al., 2020). Among the measures to reduce contagion, physical distancing was the one that perhaps had the most impact on mental health, with reports of isolation, fear of contagion and death, anxiety, depression, post-traumatic stress disorder, substance abuse, a wide range of other mental and behavioral disorders, gender or domestic violence, and child maltreatment (Bueno-Notivol et al., 2021; Garfin et al., 2020; González-Ramírez et al., 2020, Taquet et al., 2021). This raises the importance of the contributions made by psychology in the media to promote mental health, since professionals in psychology, as part of the scientific community, can be perceived as reliable and can contribute knowledge to reduce the negative impact of the crisis on mental health (Lunn et al., 2020).

The information generated by professionals in psychology or related fields undoubtedly passes through the media, which becomes a primary source of information during a crisis. Professionals in psychology should guide the content disseminated, and the media should replicate it to large audiences when discussing mental health promotion. This content should be clear and effective to avoid further harm to the population and reduce audience overexposure (Thompson et al., 2017).

### Role of media and news framing

Most of the information from psychology on dealing with the crisis was disseminated through the mass media. This raises the question as to how that information is framed by the media. The purpose of the present study was to analyze the framing of psychology in the news during the first months of the COVID-19 pandemic. News framing is conceptualized as a pattern of media coverage in which certain aspects of a topic are highlighted over others (Lecheler et al., 2015). It comprises how words, expressions, or images are highlighted and selected to expose a particular angle of the issue discussed. Empirical evidence shows that media framing has an effect on the processes of reception and impact of information (de los Santos & Nabi, 2019; Hernández-Santaolalla, 2019; Kapuściński & Richards, 2018; Lecheler & de Vreese, 2015, Igartua et al., 2012). News framing not only influences the definition of social issues but also the construction of social reality (Gui, 2021; Igartua et al., 2012). This process is generally referred to as the “framing effect”, and its consequences can be seen at an individual or societal level. The framing effect appears in small or large alterations in information processing, opinion-shaping, attitude activation and behavior, political socialization, and collective actions (Lecheler & de Vreese, 2019; Lecheler et al., 2015; Rincón-Unigarro et al., 2020).

At an individual level, frames help people make sense of the issues as a cognitive structure affecting them in terms of issue interpretation, political issues, cognitive complexity, issue support, and voter mobilization (Lecheler & de Vreese, 2019). Three cognitive processes are likely to mediate the effect of framing. Firstly, framing makes certain considerations more accessible and thus more salient. This change is more plausible as a pre-process of news framing effect (de Vreese, 2009). Secondly, belief importance changes where framing increases the weight of the beliefs it evokes rather than making them more salient. Thirdly, belief content can change when frames add new beliefs for the individual, although this effect depends on the receivers (Lecheler & de Vreese, 2019). Nonetheless, framing is often considered to have different effects and combine intermediary paths of belief importance and belief content change (Slothuus, 2008). Furthermore, it has been found that discrete emotions can be moderators of the framing effect, whereby depending on the emotion evoked, they may mobilize citizens to support or dismiss a news frame (Lerner & Keltner, 2001; Aarøe, 2011).

In the case of the COVID-19 crisis, news framing can be particularly important due to its influence on the perception of a health crisis and social roles or identities in the fight against the spread, factors that ultimately evoke actions (Gui, 2021). An experiment by Valenzuela et al. (2021) provides an example of the media framing effect during the COVID-19 crisis. When the framing of news about COVID-19 on Facebook was manipulated to emphasize economic vs. public health aspects, it influenced the level of support for restrictive measures, with support for these measures being lower in the sample of participants exposed to an economic frame.

The present study analyzes the media framing on psychology and mental health during the first months of the public health crisis associated with COVID-19 and specifically during the confinement measure. The news content published by the media concerning psychology and mental health in six countries in the Americas and Europe was analyzed to identify the most recurrent topics according to the state of emergency (i.e., the level of restrictions taken) in each country, as well as the most used scientific sources (i.e., psychology professionals. associations, other experts, or none).

## Method

### Data collection

Data was collected from media in six large countries in America and Europe. While these countries are not a random or representative selection of these continents, they provide contexts that have been affected to different extents and have, therefore, taken various strategies to handle the pandemic (Yan et al., 2021). The countries selected in America were the United States (US), Brazil, and Colombia, while for Europe were the United Kingdom, Spain, and Germany. These are also in the 20 countries with the most deaths caused by COVID-19 worldwide (Johns Hopkins University, 2021).

Two main media outlets were selected based on the weekly reach offline and online published in Newman et al.’s (2020) report on Digital News per country, ensuring the media sources had high audience traffic or national circulation. Researchers reached an agreement based on news accessibility either by a low-cost subscription or free access, as well as; media emphasizing written news and long articles providing a large corpus suitable for text analysis (See Table I in supplementary material). The news pieces collected were published between March 1st and May 31st of 2020 and contained information related to the search tags referring to COVID-19: Coronavirus, covid-19/covid, pandemic, and the terms: psychology, mental health, or well-being; and their translations to Spanish (pandemia, psicología, salud mental, bienestar), Portuguese (coronavírus, pandemia, psicologia, saúde mental, bem-estar) and German (pandemie, psychologie, psychische Gesundheit, Wohlbefinden/Wohlergehen) for the corresponding countries. The search was done manually by applying the established criteria (i.e., timeframe and keywords) to each website. No other elimination criteria were used for this data. All news articles found were entered into an Excel matrix and classified according to the following variables: *Level of restriction* for each country (a) no restrictions, (b) partial restrictions, (c) confinement and (d) partial reopening), which was established from the official communications of the health authorities of the six countries;^1^ the source of publication for each country (See table I in supplementary material), the type of news (interview, chronicle, opinion articles, news articles). Additionally, the *scientific source* was identified, with the following categories: Professionals in psychology, scientific articles, official organizations of psychology, other related experts or none.

### Data analysis: Structural Topic Modelling

This study performed Structural Topic Modeling (STM) analysis to identify the most salient topics or issues in the content published by major media regarding psychology during the early COVID-19 outbreaks. STM is an inductive technique that reduces and simplifies text to reveal hidden topics that emerge from the textual corpus in an almost unsupervised and automatic way. It is based in the algorithm known as Latent Dirichlet Allocation (Blei, 2012; Roberts et al., 2014), which assumes that each document or, in this case, news article, is a mixture of topics where each word of the document belongs to a particular topic. Its objective is to identify what combination of unknown topics could produce the analyzed documents (Lindstedt, 2019; Banks et al., 2018), which means it identifies topics from the data instead of pre-establishing them.

However, STM allows the inclusion of attributes or covariates regarding the document that could reveal how these topics are discussed or their frequency. It uses a regression framework to evaluate the influence of the covariates on the content or the prevalence of certain topics in the articles (Roberts et al., 2014). STM enables researchers to analyze the link between topics and covariates and produces more consistent patterns of topic modeling (Wesslen, 2018). In this study, the covariate *Level of Restriction* can indicate the differences in the topic frequency regarding the restrictions used to mitigate COVID-19, while the covariate *Scientific Source* can suggest the differences in topical content, or the language used to discuss topics depending on the sources used by media.

The data was analyzed using the STM package (Roberts et al., 2019) which allows easily replicable analysis with voluminous textual data in R (R Core Team, 2020). This means, the matrix with the manually retrieved textual corpus and its covariates were introduced in R, the researchers followed the recommendations for textual analysis in R by Banks et al. (2018), and the STM package performed the topic modeling analysis explained above.

**Figure 1:**
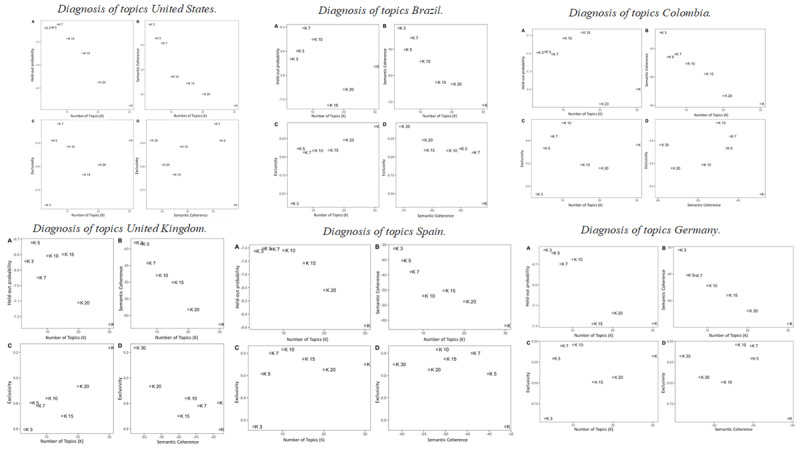
Diagnosis of topics per country. *Note*: The figure shows diagnostics for outcomes between three and 30 topics.

**Number of topics.** The number of topics was selected following different indicators between computer guidance (see ***Figure 1***) and human judgment to find balance between a significant held-out likelihood and insightful outcomes (also known as the prediction-interpretability trade-off) (Chang et al., 2009; Lindstedt, 2019; Wesslen, 2018).

***Figure 1*** shows the performance of the selected outcomes after running several ones that vary in the number of topics, between three and 30 topics (30 to 50 showed similar behavior), considering the suggestions of Lindstedt (2019) and Roberts et al. (2019) for corpus lengths not exceeding 1000 documents. Thus, we reviewed the following criteria to select the number of topics per country:

- The held-out probability, which measures the predictive power to capture natural language patterns. Values closer to zero (i.e., higher on the y-axis) indicate significantly better predictive strength. For each country, between three and 15 topics were identified as the best fit.
- The semantic coherence, which measures the average interpretation capacity of the topics by calculating the probability for a set of topic words to co-occur in the same document. Like the held-out probability, a mean value closer to zero is understood to be more interpretable than lower values. This criterion suggested running between seven and 10 topics.
- The exclusivity, which measures the probability of a word being specific to a topic. Higher scores contain a higher amount of unique vocabulary. This criterion also suggested running between seven to 10 topics.
- The balance between semantic coherence and exclusivity, this criterion proposes to choose an outcome that is not dominated by either of the two metrics. Thus, between outcomes between five and 10 topics offered the best balance for the corpus.

**Figure 2:**
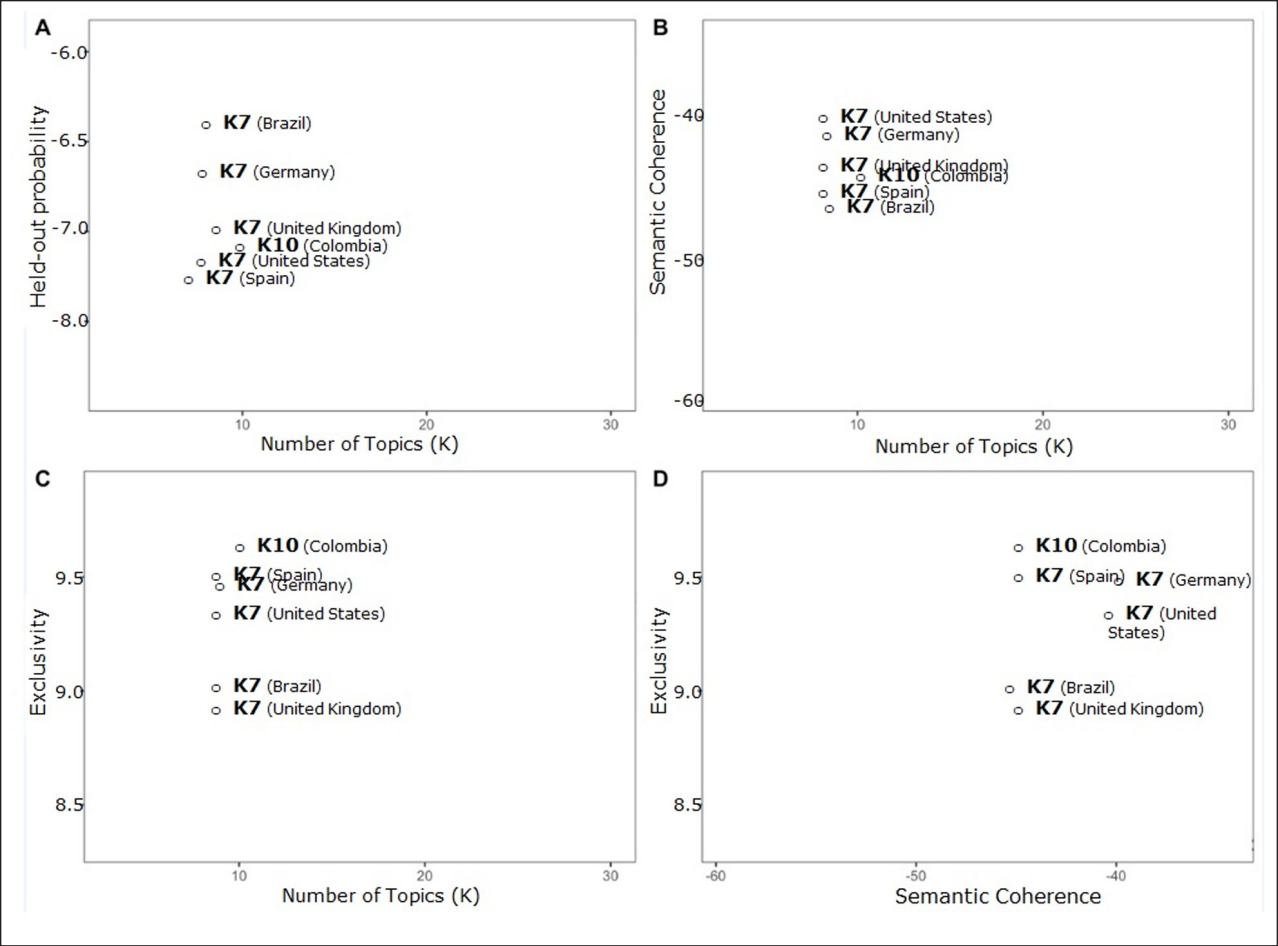
Selected Number of Topics per country. *Note*: The figure only shows the values selected by each country.

Taking into account the above criteria, as well as the expert judgment analysis of the topic content of each outcome, seven topics were selected for all countries except Colombia (See ***Figure 2***), considering them as the best outcome for each country’s content. In the case of Colombia, ten topics were a better fit due to better balance between semantic coherence and held-out probability (See ***Figure 1***), which when qualitatively assessed (by retrieving the most representative articles for the topics and the word clusters for each topic) was more interpretable than a seven or five model while holding a relevant prediction value.

## Results

### Descriptive analysis

In total, 541 items were collected from all countries. 20.33% from the United States, 18.11% from the United Kingdom, 17.74% from Spain, 16.45% from Colombia, 16.27% from Brazil, and 11.09% from Germany. In terms of the variable Level of Restriction, 51.94% news were published during Confinement, 25.87% during Partial Restrictions, 18.48% during Partial Reopening, and 3.69% in No Restrictions. Considering the data collection period (March 1 to May 1, 2020), it was not possible to collect a balanced amount of news for each level of restriction phase in the countries. The United States was the only country with news items from every level of restrictions, while in the other countries, the news articles collected were mainly from Confinement and Partial Reopening. In Brazil, almost every news item was published during Partial Restrictions, as there was no rigorous mandatory confinement applied throughout the country.

Regarding the scientific writing sources consulted by the publications, 49.54% consulted professionals in psychology, 31.24% consulted other experts, 10.17% did not use any scientific writing source, 5.55% consulted official psychology organizations, and 3.51% cited scientific articles. These results show that the most frequently used scientific writing sources were those related to psychology professionals and experts, while scientific articles and psychology organizations are less cited. Likewise, there was a low proportion of news items without scientific writing sources.

**Figure 3:**
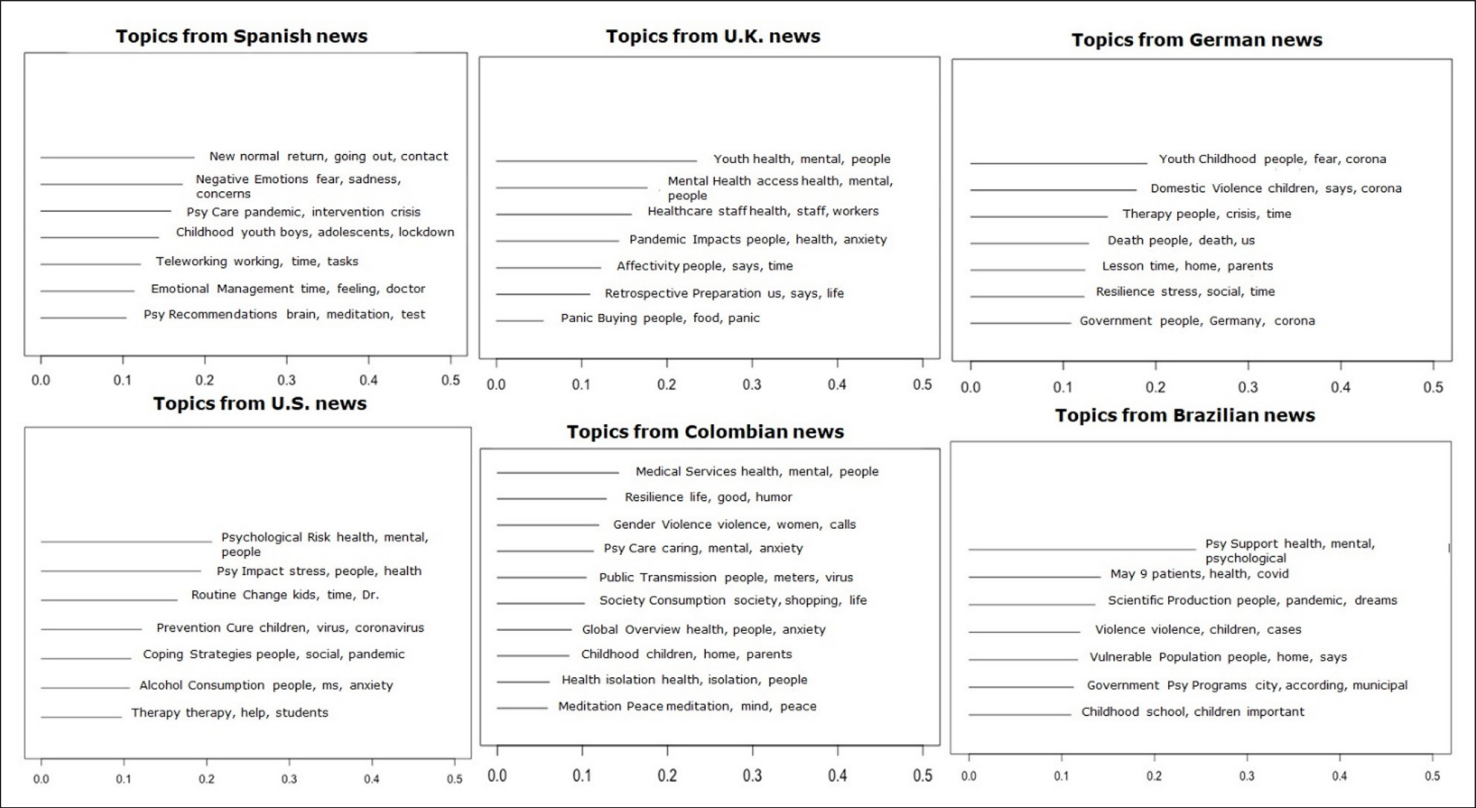
Expected topic proportion by country and the three most frequent words per topic. *Note*: All models included the covariates: Level of Restriction and Scientific Source.

**Topics.** A model was run for each country using STM, the topics per model, keywords and descriptions are displayed in ***Table 1***. When analyzing the prototype documents for each topic, we found that the topics with the highest proportion tended to address issues related to the worst aspects and consequences of mental health during the COVID-19 crisis. Although the direction of the news changed its focus group (e.g., children, young adults, healthcare workers, or women) according to the country, the psychological risk, and the need to seek mental health care as a result of the pandemic and the restrictions imposed were recurrent issues in all countries. In contrast, the lesser topics (e.g., psychological recommendations, retrospective preparation, therapy, or meditation) often related to psychological guidelines or tips for the present or future to better manage the psychological consequences of COVID-19 (see ***Figure 3***).

**Table 1:** Topic description per country with the five most frequent and exclusive words (Key Words).


COUNTRY	TOPIC	KEY WORDS	DESCRIPTION

***Germany***	Youth and childhood	Psychiatry, Adolescents, Older, Fear, Loneliness	Explanation of COVID-19 information to younger generations and the importance of not overstating the situation.

	Domestic violence	Child, Violence, Women, Domestic, Treatment	The increase in the statistics of domestic violence, with emphasis on women and children.

	Therapy	System, Traumatic, Post, Society, Crises, Actually	Psychological therapy regarding mental disorders and illnesses, as well as online consultations and practices addressing this need.

	Death	Dying, Mourners, funeral, Dead, Grief	About death and its elements such as: funerals, grief, the emotions of losing a loved one, and coping with it.

	Lesson	Learn, Learning, Failure, Start, Questions	Learning about the situation experienced and the shortcomings such as panic buying, as well as the adaptation of learning processes (Virtualization).

	Resilience	Stress, Couples, Isolations, Positive, Contacts	The opportunity to learn from the psychological stress caused by the pandemic at the personal, family and couple level.

	Government	Countries, Infections, Please, Police, Figures	Policies, decisions and regulations that are added or modified, along with official government figures and scientific information.

***Spain***	New normal	Return, Messages, Going out, Coming back, Contact, Hugs	Partial reopening scenarios regarding affectivity, social life, safety precautions, and fear.

	Negative Emotions	Circle, Look, Sadness, Syndrome, Concerns	Coping with the sadness, worry, fear, and panic caused by the pandemic and its crisis.

	Psychological Care	Scale, Association, Intervention, Public, Psychological	Psychological care for the public, students and healthcare workers.

	Childhood and youth	Boys, Adolescents, Girls, Disorders, Parents	Personal relationships between the family and the construction of affectivity.

	Teleworking	Teleworking, Tasks, Working day, Conciliate, Encourage	The massification of teleworking in the pandemic and its conciliation with other scenarios.

	Emotional Management	Doctor, Thinking, Feeling, Vulnerable, Managing	Expert recommendations to address feelings of vulnerability and expectations.

	Psychological Recommendations	Brain, Test, Breathing, Meditation, Posture	Recommendations to overcome the psychological effects of confinement.

***United Kingdom***	Youth	Young, Players, Sport, Parents, Impact	Impact on the mental health of children and youth due to COVID-19 and the alliance of sports organizations for mental health support.

	Mental health access	Psychiatrists, Welsh, Discharged, Services, Board	Impact of COVID-19 on the access to mental health services and the government’s published plans for psychological care.

	Healthcare staff	Workers, Staff, PPE, Nurses, Healthcare	Healthcare workers as the first-line of action against COVID-19, and their protection and equipment.

	Pandemic Impacts	Unemployment, Effects, Rise, SARS, Disease	The economic impact, the comparison with other pandemics and research on behavioral and psychological effects of COVID-19.

	Affectivity	Dreams, Touch, Dream, Hug, Therapy	Family and personal affection, along with the behavior of parents and children as well as contact with other relatives.

	Retrospective preparation	Prepare, Contagion, MP, Try, Today	Preparing for the future based on past and historical situations to cope with the crisis.

	Panic buying	Shaming, Market, Buying, Food, Panic	Panic buying caused by fear of shortage before confinement or other restrictions and is perceived as disruptive and embarrassing behavior.

***United States***	Psychological risk	Mind, Suicide, Centers, Touching, Doctors	Medical perspective of syndromes and symptoms that lead to depression, suicide and its prevention.

	Psychological impact	Compassion, Stress, Eating, Negative, Respondents	Changes in beliefs and common behaviors, and negative feelings due to stress or trauma.

	Routine change	Dreams, Kids, Sleep, Introverts, Friends	Change of routines and schedule that affects the circadian cycle, children’s behavior and can lead to ‘burnout’

	Prevention and cure	Theories, Motivations, Vaccine, Trump, Mother	Future expectations related to the behavioral change to prevent COVID-19 and the search for a vaccine

	Coping strategies	Loneliness, Craft, Boredom, Lonely, Sales	Related to confinement and social isolation, how to cope with the consequences of the pandemic and its crisis.

	Alcohol Consumption	Grocery, Drinking, Alcohol, Stores, Drink	Changes in the purchase and consumption of alcohol, linked to its increase and risk for the population with mental health issues.

	Therapy	Therapy, Therapists, Athletes, Counseling, Students	Therapy for students and athletes who lost both financial and emotional support due to confinement.

***Colombia***	Medical services	Medicine, Patients, Professionals, Doctors, Head	Overview of medical services during the health crisis due to COVID-19.

	Resilience	Silence, Good, Humor, Intelligence, Together	Resilience to overcome the context of crisis due to the pandemic.

	Gender violence	Women, Necessary, Violence, Calls, Sector	The increase in gender-based violence because of confinement.

	Psychological care	Caring, Communicating, Irritability, Leisure, Students	Recommendations for psychological care during confinement.

	Public transmission	Transmission, Behavior, Meters, Communities, Attitudes	Risks of virus transmission in public areas.

	Society and consumption	Society, Shopping, Death, Boredom, Dying	Consumption habits and the impact on society during the pandemic.

	Global Overview	Living, Pandemics, Environment, Information, USA	Overview of COVID-19’s evolution in other countries.

	Childhood	Parents, School, Confinement, Children, Psychologist	Management of children, education, and their care in confinement.

	Health and isolation	Patients, Ministry, Sleep, Overcrowding, Assures	Impact of social isolation on physical and psychological health.

	Meditation and peace	Peace, Meditation, Mind, Happy, Waiting	Recommendations for dealing with stress and fear during the pandemic.

***Brazil***	Psychological support	Mental, Psychological, Volunteers, Emotional, Project	Free online psychological services offered for pandemic-related psychological care (Depression, anxiety).

	May 9	Mother’s, ICU, Hospital, Patient, Nurse	How Mother’s Day is lived during the pandemic and ICU patient virtual tours.

	Scientific production	Dreams, Research, Study, University, Suicide	Scientific production related to COVID-19, such as protocols and guidelines, its effect on dreams, and the use of art for emotional expression.

	Violence	Sexual, Abuse, Violence, Police, Adolescents	Domestic and gender violence: Increase in child sexual abuse cases due to confinement and the campaign to report abuse.

	Vulnerable population	Old, Dogs, House, Elderly, Everything	Consequences of behavioral and routine changes affecting vulnerable populations (e.g., elderly and visually impaired population).

	Government Psychological Programs	City, Municipal, Municipality, hall, suspended	Online tools that connect psychologists with patients in the ICU

	Childhood	School, Pregnant, Students, Classes, Teachers	Consequences of the closure of educational centers, affecting teaching. Also, the risk of the pandemic in pregnant women.


*Note*: *Topics are displayed (per row) by each country’s model and are organized by their prevalence from highest to lowest, this is the topic’s likelihood of appearing in the news of each country (e.g., Germany model: Highest topic: Youth and childhood. Lowest: Government)*. The second column indicates the name inferred by the researchers. For this inferential task, an expert judgment was made among the researchers, considering the review of the literature cited in the introduction section and following the recommendations of Banks et al. (2018) protocol, arriving at the names in the table unanimously. The third column lists the five most important keywords, that is, the words weighted by topic-word probability, emphasizing the most frequent and exclusive of each topic. The fourth column shows an operational description of the topic built by the researchers from the keywords and the documents (i.e., news) that are highly associated with each topic.

In general, news articles in all countries have topics that raise awareness on the effects of COVID-19 on mental health, provide explanations of the events, and promote a behavioral change to mitigate either the spread of the virus or the mental stress caused by it. Results showed at least five issues shared by three or more countries and no specific differences between continents.

First, Germany, United Kingdom, and Spain emphasize the youngest (youth and children, most prevalent topics) as an audience to whom explanations of the situation may ease the impact of the measures taken. News topics regarding this population discuss the mental health impact, how to communicate this event to the youngest without overstating or provoking irrational fear, and the changes in family relationships and emotional development. This issue is also present but in lower proportions in Colombia and Brazil.

Second, these European countries also presented, in a significant proportion, information concerning access to mental health care, which changed to online consultations and practices to continue offering its services. This issue is also Brazil’s most prevalent topic, whereas in the United States it is the smallest topic, and in Colombia, no topic mentions mental health care access.

Third, although all countries have topics related to negative emotions, its proportion varies. In the United Kingdom, only one topic (affectivity) uses emotional language when discussing family and personal affection and COVID-19 emotional impacts in daily life. A common issue in the rest of the countries is the resilience or emotion regulation strategies needed to overcome this traumatic event, which entails dealing with stress, death, grief, sadness, fear, worry, feelings of vulnerability, loneliness, irritability, trauma, and emotional states related to depression and anxiety (see ***Table 1***, topics: therapy, resilience, negative emotions, emotional management, psychological impact, coping strategies, psychological care, meditation and peace, psychological support).

Fourth, another salient issue is violence against women and children in Germany, Colombia, and Brazil, where topics highlight the increase in cases of domestic and gender violence. These topics illustrate how the strict lockdown and the inability to leave home unless necessary may have affected the care programs and services for victims of domestic violence.

Finally, all countries except Germany underline a behavioral change that has been caused or increased either by fear and similar emotions (panic, worry, stress) or due to social isolation measures. These changes involve consumer habits like panic buying in the United Kingdom or an increase in alcohol purchase in the United States; home and family routines affected by working/studying from home in Spain and the United States; how these routines changes affect the vulnerable population in Brazil; as well as preventive behavior (masks, social distance, testing, and others) in public spaces in Colombia, Spain, and the United States.

### Influence of covariates

**Compared effects by Level of Restriction.** We analyzed the influence of the covariate Level of Restriction through STM. During the phase with no restrictions, no topic was particularly representative or salient in the countries analyzed. This result may be at least in part due to the low amount of news collected, which only represents 3.69% of the text corpus. Furthermore, during this early period of the pandemic, the media’s focus was on the expansion of the virus rather than its psychological consequences.

During the Partial Restrictions^2^ phase, when the first trade and mobility restrictions began, the covariate influenced the topics in the United Kingdom, Spain, and the United States (see ***Figure 3***), which focused on two main news frames. The first one relates to a) the social behaviors that emerged in response to the crisis. For instance, teleworking as a solution that decreases social contact leads to drastic changes in the routine and care of family and home. Likewise, the panic buying that appears as a reaction to the unpredictability of the environment, behavior later perceived as disruptive or shameful. The second frame focuses on b) psychological care in terms of access to mental health services and the psychological impact of the new limited context, and the restrictions of the physical and social interactions.

The confinement phase was the most restrictive period due to the shutdown of all non-essential trade and mobility, which resulted in all the government and media attention focused on COVID-19 and its consequences. Half of the news collected were published during this period. As in the Partial Restrictions phase, the publications aimed to offer psychological care services and to raise awareness about the psychological effects of confinement and social isolation. However, because of the constraining context, there are also topics focused on mitigating its impact and generating behavioral change (e.g., Coping strategies, Care, Psychological recommendations, Childhood).

**Figure 4:**
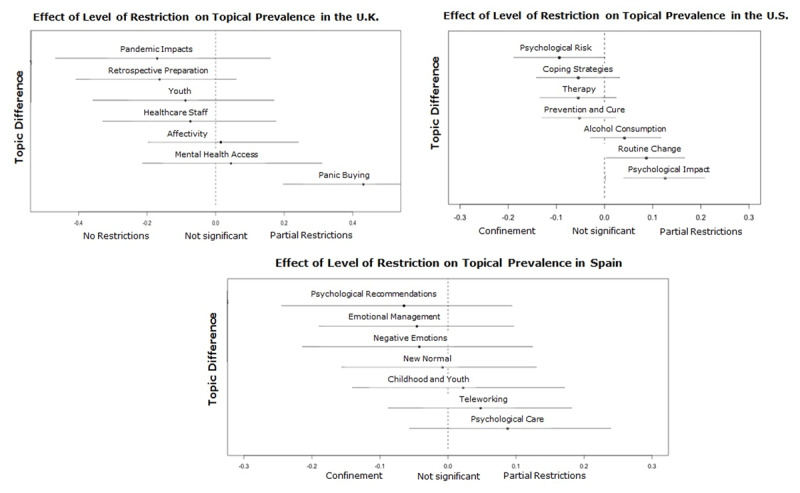
Effect of the covariate Level of Restriction on the topics of United Kingdom, United States, and Spain.

**Figure 5:**
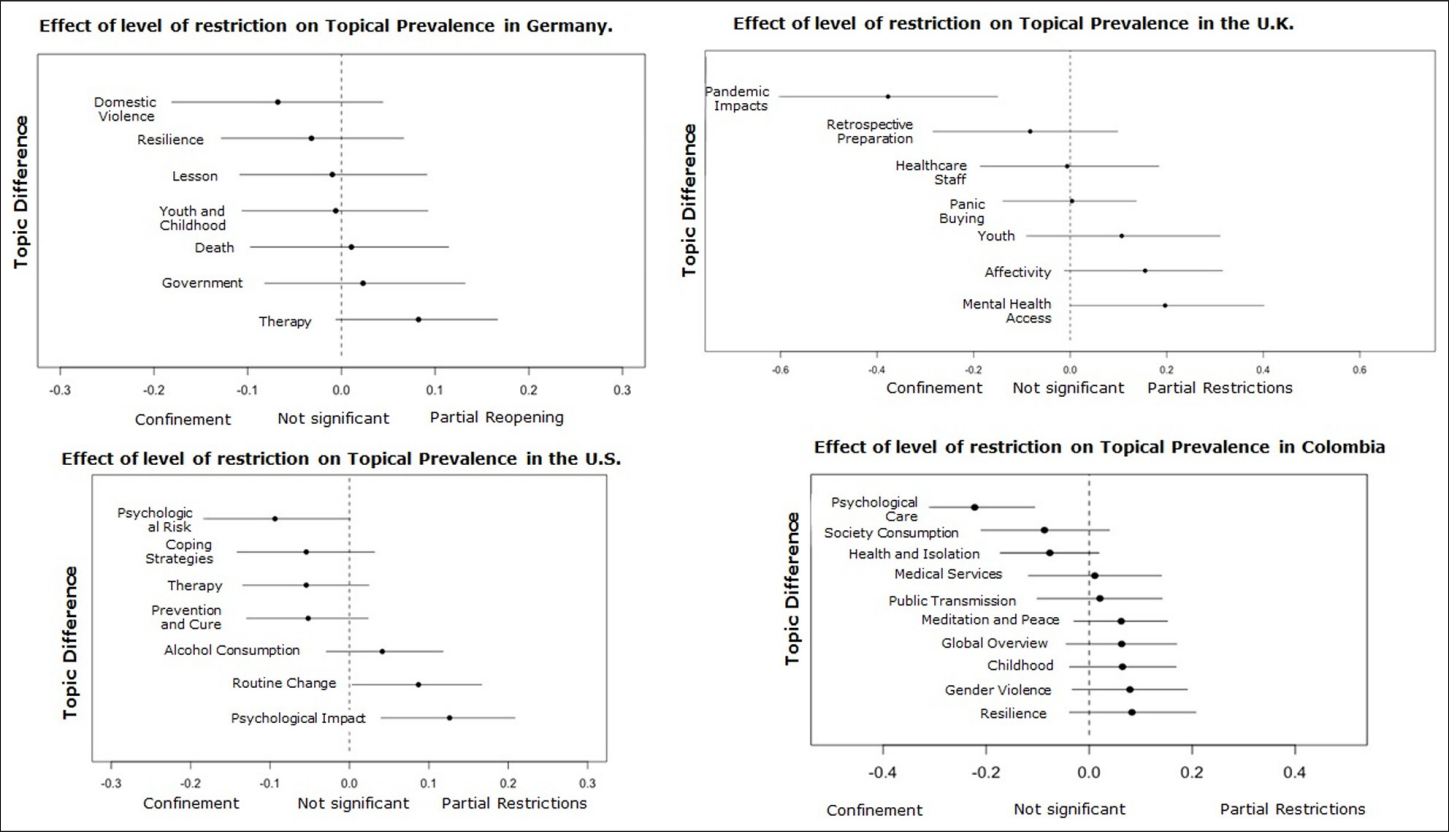
Effect of the covariate Level of Restriction on the topics of Germany, United Kingdom, United States, and Colombia.

As a consequence of the mobility limitation, in Germany, the visibility of domestic violence increased, while in Colombia, the concern was the management of children and their adaptation to the schools’ closure. In the United States, Colombia, and Spain, the topics related to mental health and psychological aspects increased their prevalence and variety, responding to the difficulty of what this crisis meant (See ***Figures 4*** and ***5***). It is worth noting that in the case of the United Kingdom, all of the topics increased their prevalence during this phase, which may be because the majority of its collected news items were from this period.

**Figure 6:**
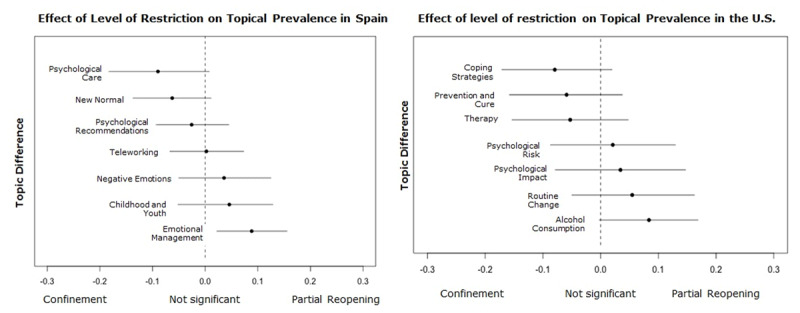
Effect of the covariate Level of Restriction on the topics of Spain and the United States.

The fourth and last phase, Partial Reopening, is characterized by the uncertainty between the expectations by the economic and social life reactivation and the fear of still being able to control the contagion curve. The influence of this phase on the prevalence of the topics is visible in Germany, Spain, the United States, and Colombia, where the two news frames identified before are present a) Social behaviors as a response to the crisis and b) Psychological care. Regarding the first news frame, topics focused on creating awareness of specific problems caused by the confinement in the United States and Colombia: Alcohol consumption and Gender violence. In the second news frame, media discussed the offer of therapy, its availability, and new forms of access, as well as the experts’ recommendations regarding the feelings of vulnerability and the expectations of a new global scenario (e.g., Therapy, Emotional Management) (See ***Figures 5*** and ***6***).

**Compared effects by Scientific Source.** As with the covariate Level of Restriction, we analyzed through STM the influence of the covariate Scientific source on the prevalence of the topics. This analysis identified that the topic prevalence varied according, mainly, to the consultation of professionals in psychology and other experts.

Those publications that consulted Professionals in psychology discussed more frequently a) access to mental health services, care, and psychological risk alongside b) social issues such as domestic, gender-based violence, and the changes and challenges caused by teleworking. These news frames and their topics were generally addressed by professionals in psychology to generate awareness about the particular problem or to encourage a behavioral change.

Particularly in the United States, the most prevalent topic (Psychological risk) was the most frequent during confinement and when the source was a professional in psychology. In this country, the media consulted these professionals to discuss future expectations related to the behavioral change to prevent transmission. Likewise, in Spain, consultation with professionals in psychology was more prevalent when news content focused on modification or adaptation of behavior to new ways of interaction and work. Lastly, in Brazil, the most prevalent topic (Psychological support) was not affected by the type of scientific source used despite care being one of the main areas of psychological practice.

**Figure 7:**
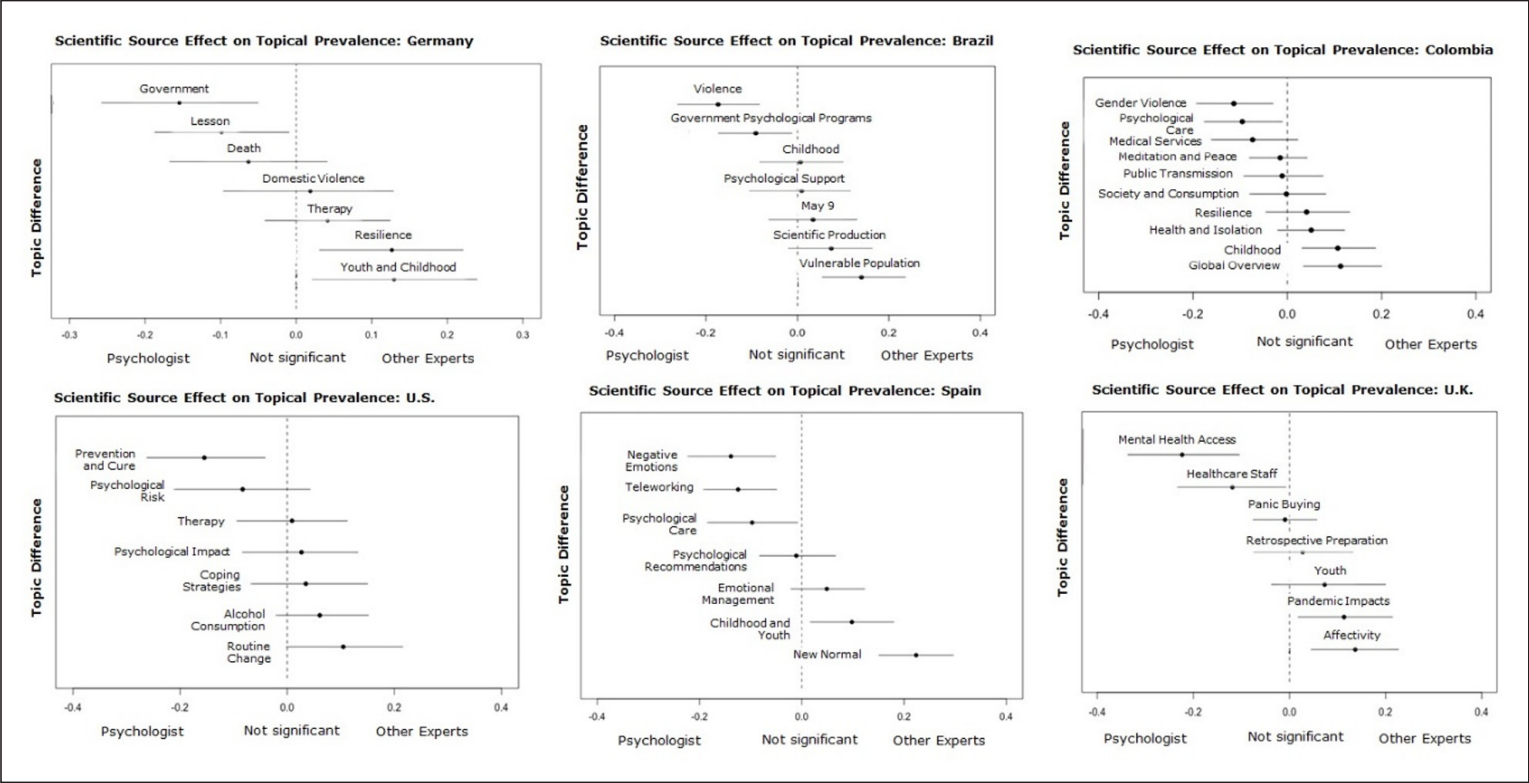
Effect of consulting Professionals in psychology and Other experts as scientific source on the topics of each country.

The use of “Other Experts” as scientific sources also affected the prevalence of the topics in all countries (see ***Figure 7***). Unlike the news frames present when the source consulted was a professional in psychology, professionals in other areas did not approach the psychological field from the point of view of care and access to mental health services, but rather dealt with topics related to the life cycle, for example, youth and childhood in Germany, United Kingdom, Spain, and Colombia. In these countries, the media seemed to prefer various professionals to discuss personal and family issues that are affected by the health crisis. These experts are the preferred media source when giving general explanations about social phenomena (e.g., topics Routine Change, New Normal, and Global Overview).

Furthermore, in Germany, the media consulted professionals in psychology for the topic Government, which is related to its decisions and regulations, and the scientific information to be published. Meanwhile, when the source was other experts, topics with a more mainstream psychology approach were more prevalent, such as overcoming psychological stress within family or couple, or personally (e.g., topic Resilience).

**Figure 8:**
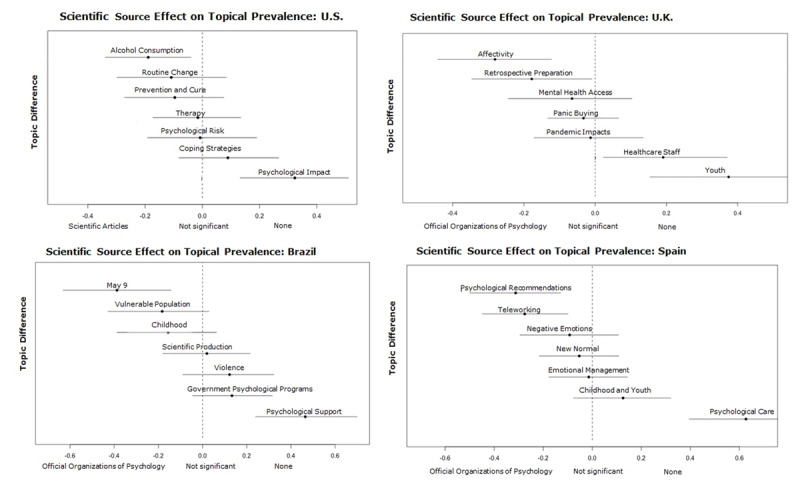
Effect of no scientific source (None) on the topics of the United States, United Kingdom, Brazil and Spain.

Finally, although the amount of news that had no scientific source (“None”) is 10.17%, it is worth noting that there was a tendency to address topics such as the psychological impact on the population and youth, psychological support, and care. This result is most evident in the United States, Brazil, Spain, and the United Kingdom (See ***Figure 8***).

## Discussion

This article analyzed the media framing of the information transmitted regarding psychology and mental health in the first months of the COVID-19 crisis in different countries of America and Europe. More specifically, it examined whether the media framing varied according to (a) the level of restriction during publication or (b) the scientific source used to generate the content.

The findings indicate that the media in these countries highlight the importance of mental health during the COVID-19 crisis. Overall, there was a strong emphasis on reporting the risks and psychological consequences of the pandemic at all levels of restriction. These news frames generally sought to influence the perception of risk and the need to seek psychological care. In this regard, our findings suggest that these frames focused, depending on each country’s needs, on specific demographic groups and available control measures for each situation, as evidenced by Bish & Michie (2010) in the media coverage of the H1N1 health crisis.

In all countries, news articles raised awareness and reported on events related to COVID-19 and mental health. This suggests that the news frames aimed to help the public to interpret the events and make decisions according to their context, either promoting preventive individual behavior or explaining social changes that mitigate the spread. The way in which the media framed the information related to psychological aspects showed an effort to translate the scientific data and turn it into a useful tool to face the crisis. Examples of this are the increase of gender-based or domestic violence, the problems related to youth and children, and the behavioral changes due to fear or social isolation. Based on the credibility of the sources and the empathic approach, these news items promoted individual actions to mitigate the phenomenon, in line with the variables required for effective communication (Lunn et al., 2020).

The findings also indicate that the prevalence of topics varied according to the restriction level and the scientific source used. As for the variable *level of restriction*, there was a change in the news’ focus, prioritizing certain events based on the context and the population’s needs. Although the psychological risk is present at all levels of restriction, it is more prevalent in partial restrictions and confinement. As such, these results show a rapid response from the media to new contexts emerging from the pandemic and its crisis. Topics such as teleworking or panic buying are more prevalent in the partial restrictions in the United Kingdom, Spain, and the United States, where information on the form of containment of the virus was unclear due to its rapid spread.

During the confinement, the countries differed in the problems that most afflict them. In Germany, for example, domestic violence became a prevalent issue, while in Colombia, the adaptation of youth to school closure fostered a concern due to the parents’ unavailability to care for them during the working day. These differences regarding the level of restriction show that the media relate social behaviors and changes to psychology but could also have benefited from psychology as a relevant social science in the prevention of the spread of the virus, by promoting preventive behavior during the early stages of the pandemic.

The news frames found in the variable *scientific source* show two different approaches to the psychological aspects of the health crisis. On the one hand, the news frame of psychology professionals focused on raising awareness about psychological care, access to mental health services, and its importance facing the COVID-19 crisis. Those news articles that consulted psychologists as a source covered topics of specific problems with the aim of generating a behavioral change seeking to mitigate the responses of stress, worry, and fear as previously suggested by Garfin et al. (2020). On the other hand, the news items where other experts were consulted tended to provide information that generated explanations to social phenomena, covering not only the psychological issues at an individual level but building a multidisciplinary explanatory framework.

During the early stage of the pandemic, psychology’s presence in the media mainly involved a reaction to the challenge by primarily dealing with mental health, protection of youth and children, emotion regulation strategies, and risky as well as preventive behavioral changes. The scientific knowledge of psychologists provided to the media responded to the challenges of dealing with COVID-19 due to lockdowns and social distancing with low to no physical contact in public spaces. This psychological expertise is related to an individual and interpersonal level, like dealing with emotions and relationships (among family, friends, and other social circles). In contrast, not much space is given to social scientists’ capacity to address macro-social concerns, such as the prejudice to specific population groups or the process of dehumanization generated by the pandemic (Bavel et al., 2020). However, the media did relate psychology to social behavior and changes, which could have enabled more space for psychologists to explain traumatic events, collective actions, or beliefs.

Even though professionals in psychology have taken various actions through different channels to help the population overcome this traumatic event, such as participation in health policies, increasing accessibility of psychological services, providing psychoeducation, improving access to key information through online channels, and offering care to specific and more vulnerable populations (de Almondes et al., 2021), only a narrow portion of this work is visible through the media.

Finally, we recognize the various limitations of this study. First, the time limit of the data collection makes it impossible to have a global view of the media framing during the pandemic development throughout the year 2020, due to its focus on the first months of the spread and mobility restrictions in the selected countries. Secondly, we recognize that the uneven number of news items gathered at every level of restriction limited the covariate contrast in some analyzed countries. Thirdly, the used media do not necessarily represent the news framing of all the existent outlets in each country, but rather an approach to addressing COVID-19 and mental health. Fourthly, this study analyzed only traditional media and could have included social media content. However, newspapers are still considered by many people as their main source of information, and perceived as the most reliable, when it comes to the consumption of hard news (e.g., politics, business, economics, and finance) (Fan et al., 2021). Similarly, many news outlets have migrated to the internet (as is the case for the selected media), to online spaces where they adapt the content to the format of social media to have greater reach, becoming an important consumption scenario (Lázaro-Rodríguez & Herrera-Viedma, 2020; Welbers & Opgenhaffen, 2019). Finally, as in the case of the COVID-19 pandemic, evidence shows that the media have been especially important in generating effective social responses (Krawczyk et al., 2021).

## Transparency statement

The authors affirm that this manuscript is an accurate and transparent report of the study. No important aspects of the study have been omitted.

## Data accessibility statement

Supporting information of this article (databases, R packages employed for data analysis and R code) are publicly available at: *https://osf.io/ewfrb/?view_only=d312717027c04e7c930adb13ac304d83*.

## Additional File

The additional file for this article can be found as follows:

10.5334/pb.1054.s1 Supplementary File 1. Table I. Media by Country.

## Footnotes

The timeframes for the categories of the variable Level of Restriction were built based on the official communications of each country regarding their management of COVID-19 during the established period: **a) United States**: *a)* March 1^st-^ 15^th^, *b)* March 16^th-^ April 2^nd^, *c)* April 3^rd^ – May 15^st^, *d)* March 16^th^ – May 31^st^ **b) Brazil**: *a)* March 1^st^-20^th^, *b)* March 21^st^ – May 31^st^, *c)* NA, *d)* NA; **c) Colombia**: *a)* March 1^st^- 11^th^, *b)* March 12^th^ – 23^rd^, *c)* March 24^th^ – May 4^th^, *d)* May 5^th^ -31^st^ ; **d) United Kingdom**: *a)* March 1^st^ -17^th^, *b)* March 18^th^ -23^rd^, *c)* March 24^th^ – March 31^st^, *d)* April 1^st^ – May 31^st^ ; **e) Spain**: *a)* March 1^st^ -7^th^, *b)* March 8^th^ -13^th^, *c)* March 14^th^ – May 10^th^, *d)* May 11^th^ – 31^st^; **f) Germany**: *a)* March 1^st^ – 15th, *b)* March 16^th^ – 21^st^, *c)* March 22^nd^ – April 19^th^, *d)* April 20^th^ – May 31^st^.

It is worth mentioning that in Germany, there was not enough news to carry out an analysis of the Partial Restrictions phase. Whereas in Brazil, no comparisons were made between the phases of *Level of restriction* as all news were coded as Partial Restrictions.

## References

[1] Aarøe, L. (2011). Investigating Frame Strength: The Case of Episodic and Thematic Frames. Political Communication, 28(2), 207–226. DOI: 10.1080/10584609.2011.568041

[2] Banks, G. C., Woznyj, H. M., Wesslen, R. S., & Ross, R. L. (2018). A Review of Best Practice Recommendations for Text Analysis in R (and a User-Friendly App). Journal of Business and Psychology, 33(4), 445–459. DOI: 10.1007/s10869-017-9528-3

[3] Bavel, J. J. V., Baicker, K., Boggio, P. S., Capraro, V., Cichocka, A., Cikara, M., Crockett, M. J., Crum, A. J., Douglas, K. M., Druckman, J. N., Drury, J., Dube, O., Ellemers, N., Finkel, E. J., Fowler, J. H., Gelfand, M., Han, S., Haslam, S. A., Jetten, J., … Willer, R. (2020). Using social and behavioural science to support COVID-19 pandemic response. Natural Human Behaviour, 4, 460–471. DOI: 10.1038/s41562-020-0884-z 32355299

[4] Bicchieri, C., Fatas, E., Aldama, A., Casas, A., Deshpande, I., Lauro, M., Parilli, C., Spohn, M., Pereira, P., & Wen, R. (2021). In science we (should) Trust: Expectations and compliance across nine countries during the COVID-19 pandemic. PLOS ONE, 16(6). DOI: 10.1371/journal.pone.0252892 PMC8177647 34086823

[5] Bish, A., & Michie, S. (2010). Demographic and attitudinal determinants of protective behaviours during a pandemic: A review. British Journal of Health Psychology, 15, 797–824. DOI: 10.1348/135910710X485826 20109274 PMC7185452

[6] Blei, D. M. (2012). Probabilistic topic models. Communications of the ACM, 55(4), 77–84. DOI: 10.1145/2133806.2133826

[7] Bueno-Notivol, J., Gracia-García, P., Olaya, B., Lasheras, I., López-Antón, R., & Santabárbara, J. (2021). Prevalence of depression during the COVID-19 outbreak: A meta-analysis of community-based studies. International Journal of Clinical and Health Psychology, 21(1), 100196. DOI: 10.1016/j.ijchp.2020.07.007 32904715 PMC7458054

[8] Chang, J., Gerrish, S., Wang, C., Boyd-Graber, J. L., & Blei, D. M. (2009). Reading tea leaves: How humans interpret topic models. In Y. Bengio, D. Schuurmans, J. D. Lafferty, C. K. I. Williams & A. Culotta (Eds.), Advances in neural information processing systems (pp. 288–296). Curran Associates. https://proceedings.neurips.cc/paper/2009/file/f92586a25bb3145facd64ab20fd554ff-Paper.pdf

[9] de Almondes, K. M., Bizarro, L., Miyazaki, M., Soares, M., Peuker, A. C., Teodoro, M., Modesto, J. G., Veraksa, A. N., Singh, P., Han, B., & Sodi, T. (2021). Comparative Analysis of Psychology Responding to COVID-19 Pandemic in Brics Nations. Frontiers in Psychology, 12, 567585. DOI: 10.3389/fpsyg.2021.567585 34149490 PMC8210845

[10] de los Santos, T. M., & Nabi, R. L. (2019). Emotionally charged: Exploring the role of emotion in online news information seeking and processing. Journal of Broadcasting & Electronic Media, 63(1), 39–58. DOI: 10.1080/08838151.2019.1566861

[11] de Vreese, C. H. (2009). Framing the economy: Effects of journalistic news frames. In P. D’Angelo & J. A. Kuypers (Eds.), Doing news framing analysis. Empirical and theoretical perspectives (pp. 187–214). New York: Routledge.

[12] Fan, B., Liu, S., Pei, G., Wu, Y., & Zhu, L. (2021). Why Do You Trust News? The Event-Related Potential Evidence of Media Channel and News Type. Frontiers in Psychology, 12(April), 1–9. DOI: 10.3389/fpsyg.2021.663485 PMC8081029 33935924

[13] Garfin, D. R., Silver, R. C., & Holman, E. A. (2020). The novel coronavirus (COVID-2019) outbreak: Amplification of public health consequences by media exposure. Health Psychology, 39(5), 355–357. DOI: 10.1037/hea0000875 32202824 PMC7735659

[14] González-Ramírez, M. T., QuezadaBerumen, L. Q., & Landero Hernández, R. (2020). Longitudinal study of the psychological impact of the contingency response to COVID-19 in Mexico. Universitas Psychologica, 19, 1–10. DOI: 10.11144/Javeriana.upsy19.lspi

[15] Gui, L. (2021). Media framing of fighting COVID-19 in China. Sociology of Health & Illness, 43(4), 966–970. DOI: 10.1111/1467-9566.13271 33782963 PMC8251264

[16] Hernández-Santaolalla, V. (2019). Los efectos de los medios de comunicación de masas. Editorial UOC. DOI: 10.12795/Comunicacion.2018.i16.09

[17] Igartua, J. J., Moral-Toranzo, F., & Fernández, I. (2012). Cognitive, attitudinal, and emotional effects of news frame and group cues, on processing news about immigration. Journal of Media Psychology, 23(1), 174–185. DOI: 10.1027/1864-1105/a000050

[18] Johns Hopkins University. (2021). COVID-19 Dashboard by the Center for Systems Science and Engineering (CSSE). Coronavirus Resource Center. Retrieved September 24, 2021, from https://coronavirus.jhu.edu/map.html

[19] Kapuściński, G., & Richards, B. (2018). Destination risk news framing effects–the power of audiences. The Service Industries Journal, 42, 107–130. DOI: 10.1080/02642069.2018.1441402

[20] Krawczyk, K., Chelkowski, T., Laydon, D. J., Mishra, S., Xifara, D., Flaxman, S., Flaxman, S., Mellan, T., Schwämmle, V., Röttger, R., Hadsund, J. T., & Bhatt, S. (2021). Quantifying online news media coverage of the COVID-19 pandemic: Text mining study and resource. Journal of Medical Internet Research, 23(6), 1–16. DOI: 10.2196/28253 PMC8174556 33900934

[21] Lázaro-Rodríguez, P., & Herrera-Viedma, E. (2020). Noticias sobre Covid-19 y 2019-nCoV en medios de comunicación de España: el papel de los medios digitales en tiempos de confinamiento. El profesional de la información, 29(3). DOI: 10.3145/epi.2020.may.02

[22] Lecheler, S., Bos, L., & Vliegenthart, R. (2015). The Mediating Role of Emotions: News Framing Effects on Opinions About Immigration. Journalism & Mass Communication Quarterly, 92(4), 812–838. DOI: 10.1177/1077699015596338

[23] Lecheler, S., & de Vreese, C. H. (2015). How Long Do News Framing Effects Last? A Systematic review of Longitudinal Studies. Annals of the International Communication Association, 40(1), 3–30. DOI: 10.1080/23808985.2015.11735254

[24] Lecheler, S., & de Vreese, C. H. (2019). News framing effects. Taylor & Francis. DOI: 10.4324/9781315208077

[25] Lerner, J. S., & Keltner, D. (2001). Fear, anger, and risk. Journal of Personality and Social Psychology, 81(1), 146–159. DOI: 10.1037/0022-3514.81.1.146 11474720

[26] Lindstedt, N. C. (2019). Structural Topic Modeling For Social Scientists: A Brief Case Study with Social Movement Studies Literature, 2005–2017. Social Currents, 6(4), 307–318. DOI: 10.1177/2329496519846505

[27] López-López, W. (2020). La pandemia del coronavirus y las deficiencias de la comunicación científica global. Journals and Authors Solutions Blog. DOI: 10.25012/blog.16.03.2020

[28] Lunn, P. D., Belton, C. A., Lavin, C., McGowan, F. P., Timmons, S., & Robertson, D. A. (2020). Using Behavioral Science to help fight the Coronavirus. Journal of Behavioral Public Administration, 3(1). DOI: 10.30636/jbpa.31.147

[29] Masip, P., Aran-Ramspott, S., Ruiz-Caballero, C., Suau, J., Almenar, E., & Puertas-Graell, D. (2020). Consumo informativo y cobertura mediática durante el confinamiento por el Covid-19: sobreinformación, sesgo ideológico y sensacionalismo. El profesional de la información, 29, 3. DOI: 10.3145/epi.2020.may.12

[30] Moya, M., Willis, G. B., Paez, D., Pérez, J. A., Gómez, Á., Sabucedo, J. M., … Salanova, M. (2020). La Psicología Social ante el COVID-19: Monográfico del International Journal of Social Psychology (Revista de Psicología Social). DOI: 10.31234/osf.io/fdn32

[31] Newman, N., Fletcher, R., Schulz, A., Andi, S., & Nielsen, R. K. (2020). Reuters Institute Digital News Report 2020 (pp. 1–109). Oxford: University of Oxford. https://reutersinstitute.politics.ox.ac.uk/sites/default/files/2020-06/DNR_2020_FINAL.pdf

[32] Ng, R., Chow, T. Y., & Yang, W. (2021). News media narratives of covid-19 across 20 countries: Early global convergence and later regional divergence. PLOS ONE, 16(9). DOI: 10.1371/journal.pone.0256358 PMC8409659 34469446

[33] R Core Team. (2020). R: A Language and Environment for Statistical Computing. Retrieved from https://www.r-project.org/

[34] Rincón-Unigarro, C., Correa-Chica, A., López-López, W., Morales-Sierra, M. P., & Rivera-Escobar, S. (2020). Encuadres Mediáticos del Perdón y la Reconciliación en el Contexto del Conflicto Armado Colombiano. Revista Colombiana de Psicología, 29(1), 105–123. DOI: 10.15446/.v29n1.81505

[35] Roberts, M., Stewart, B., & Tingley, D. (2019). stm: An R Package for Structural Topic Models. Journal of Statistical Software, 91(2), 1–40. DOI: 10.18637/jss.v091.i02

[36] Roberts, M., Stewart, B., Tingley, D., Lucas, C., Leder‐Luis, J., Kushner Gadarian, S., Albertson, B., & Rand, D. (2014). Structural Topic Models for Open‐Ended Survey Responses. American Journal of Political Science, 58(4), 1064–1082. DOI: 10.1111/ajps.12103

[37] Shullman, S., & Evans, A. (2020, March 27). Overview of APA’s actions around COVID-19. American Psychological Association. Retrieved June 30, 2020, https://www.apa.org/members/content/covid-19-actions. DOI: 10.1037/e502602020-001

[38] Slothuus, R. (2008). More than weighting cognitive importance: A dual process model of issue framing effects. Political Psychology, 29(1), 1–28. DOI: 10.1111/j.1467-9221.2007.00610.x

[39] Sturgis, P., Brunton-Smith, I., & Jackson, J. (2021). Trust in science, social consensus and vaccine confidence. Nature Human Behaviour, 5, 1528–1534. DOI: 10.1038/s41562-021-01115-7 34002053

[40] Taquet, M., Luciano, S., Geddes, J. R., & Harrison, P. J. (2021). Bidirectional associations between COVID-19 and psychiatric disorder: retrospective cohort studies of 62 354 COVID-19 cases in the USA. The Lancet Psychiatry, 8(2), 130–140. DOI: 10.1016/S2215-0366(20)30462-4 33181098 PMC7820108

[41] Thompson, R. R., Garfin, D. R., Holman, E. A., & Silver, R. C. (2017). Distress, worry, and functioning following a global health crisis: A national study of Americans’ responses to Ebola. Clinical Psychological Science, 5, 513–521. DOI: 10.1177/2167702617692030

[42] Valenzuela, S., Bachmann, I., Mujica, C., Grassau, D., Labarca, C., Halpern, D., & Puente, S. (2021). Competing Frames and Melodrama: The Effects of Facebook Posts on Policy Preferences about COVID-19. Digital Journalism, 9, 1411–1430. DOI: 10.1080/21670811.2021.1943479

[43] Welbers, K., & Opgenhaffen, M. (2019). Presenting News on Social Media: Media logic in the communication style of newspapers on Facebook. Digital Journalism, 7(1), 45–62. DOI: 10.1080/21670811.2018.1493939

[44] Wesslen, R. (2018). Computer-assisted text analysis for social science: Topic models and beyond. arXiv preprint. arXiv:1803.11045

[45] Yan, H., Zhu, Y., Gu, J., Huang, Y., Sun, H., Zhang, X., Wang, Y., Qiu, Y., & Xi Chen, S. (2021). Better strategies for containing Covid-19 PANDEMIC: A study of 25 countries via a vSIADR model. Proceedings of the Royal Society A: Mathematical, Physical and Engineering Sciences, 477(2248). DOI: 10.1098/rspa.2020.0440 PMC8300607 35153551

[46] Zheng, Y., Goh, E., & Wen, J. (2020). The effects of misleading media reports about COVID-19 on Chinese tourists’ mental health: A perspective article. Anatolia: An International Journal of Tourism and Hospitality Research, 31(2), 337–340. DOI: 10.1080/13032917.2020.1747208

