# Supplementary material for: The Role of Psychology in Media During the COVID-19 Pandemic: A Cross-National Study
Source: Psychol Belg. 2022 Apr 12;62(1):136–51. doi: 10.5334/pb.1054 (PMC9009363; doi:10.5334/pb.1054)
Supplement: Supplementary File 1. Table I. — Media by Country. [file pb-62-1-1054-s1.pdf]

Supplementary Table I

Media by country

Country	Media	Type of Media	Percentage from total sample of news
United States	The Washington Post	Newspaper	10.5%
	The New York Times	Newspaper	9.7%
Brazil	G1 de Rede Globo	Online News	15.3%
	Jornal Do Brasil	Newspaper	0.9%
Colombia	El Tiempo	Newspaper	8.8%
	El Espectador	Newspaper	7.5%
United Kingdom	The Guardian	Newspaper	10.7%
	BBC News	Online News	7.3%
Spain	El País	Newspaper	9.7%
	ABC	Newspaper	7.9%
Germany	Süddeutsche Zeitung	Newspaper	9.2%
	RTL.de	Online News	1.8%
